# Complementary water quality observations from high and medium resolution Sentinel sensors by aligning chlorophyll-*a* and turbidity algorithms

**DOI:** 10.1016/j.rse.2021.112651

**Published:** 2021-11

**Authors:** Mark A. Warren, Stefan G.H. Simis, Nick Selmes

**Affiliations:** Plymouth Marine Laboratory, Plymouth PL1 3DH, UK

## Abstract

High resolution imaging spectrometers are prerequisite to address significant data gaps in inland optical water quality monitoring. In this work, we provide a data-driven alignment of chlorophyll-*a* and turbidity derived from the Sentinel-2 MultiSpectral Imager (MSI) with corresponding Sentinel-3 Ocean and Land Colour Instrument (OLCI) products. For chlorophyll-*a* retrieval, empirical ‘ocean colour’ blue-green band ratios and a near infra-red (NIR) band ratio algorithm, as well as a semi-analytical three-band NIR-red ratio algorithm, were included in the analysis. Six million co-registrations with MSI and OLCI spanning 24 lakes across five continents were analysed. Following atmospheric correction with POLYMER, the reflectance distributions of the red and NIR bands showed close similarity between the two sensors, whereas the distribution for blue and green bands was positively skewed in the MSI results compared to OLCI. Whilst it is not possible from this analysis to determine the accuracy of reflectance retrieved with either MSI or OLCI results, optimizing water quality algorithms for MSI against those previously derived for the Envisat Medium Resolution Imaging Spectrometer (MERIS) and its follow-on OLCI, supports the wider use of MSI for aquatic applications. Chlorophyll-*a* algorithms were thus tuned for MSI against concurrent OLCI observations, resulting in significant improvements against the original algorithm coefficients. The mean absolute difference (MAD) for the blue-green band ratio algorithm decreased from 1.95 mg m^−3^ to 1.11 mg m^−3^, whilst the correlation coefficient increased from 0.61 to 0.80. For the NIR-red band ratio algorithms improvements were modest, with the MAD decreasing from 4.68 to 4.64 mg m^−3^ for the empirical red band ratio algorithm, and 3.73 to 3.67 for the semi-analytical 3-band algorithm. Three implementations of the turbidity algorithm showed improvement after tuning with the resulting distributions having reduced bias. The MAD reduced from 0.85 to 0.72, 1.22 to 1.10 and 1.93 to 1.55 FNU for the 665, 708 and 778 nm implementations respectively. However, several sources of uncertainty remain: adjacent land showed high divergence between the sensors, suggesting that high product uncertainty near land continues to be an issue for small water bodies, while it cannot be stated at this point whether MSI or OLCI results are differentially affected. The effect of spectrally wider bands of the MSI on algorithm sensitivity to chlorophyll-*a* and turbidity cannot be fully established without further availability of in situ optical measurements.

## Highlights

• globally tuned inland water quality algorithms for MSI.
• OLCI and MSI Rw from POLYMER agreed well in longer wavebands compared to shorter.
• OC2 algorithm coefficients show strong dependency on input dataset.

## 1 Introduction

Globally, there are over 100 million lakes and reservoirs (Verpoorter et al., 2014) with the vast majority not observable using the spatial resolution of current ocean colour sensors. Ensuring healthy and productive inland water bodies underpins several of the United Nations Sustainable Development Goals (SDG) and is at the core of SDG 6 on clean water. Reporting on SDG 6 and international water quality frameworks such as the European Water Framework Directive (2000/60/EC), which aim to record ambient surface water quality of inland and coastal water resources, is only feasible over large areas or hazardous regions when remote sensing is used (Papathanasopoulou et al., 2019). For climate studies, lake water-leaving reflectance (LWLR) was recently adopted in the Lakes Essential Climate Variable (ECV) by the Global Climate Observing System (GCOS 200, 2016). LWLR, or reflectance in short, describes the fate of sunlight entering the water column and allows biogeochemical quantities such as chlorophyll-*a* (chl-*a*) or water transparency, turbidity, suspended particle concentration or dissolved organic matter, to be derived from diagnostic reflectance signatures. Accurately and consistently deriving these quantities in relatively small inland water bodies is now a vital next step for the water quality remote sensing research community, with progress made in regional studies (e.g. Toming et al., 2016; Bresciani et al., 2019; Pahlevan et al., 2019).

Current satellite monitoring platforms offer unprecedented capabilities to observe the optical quality of water bodies globally (Groom et al., 2018). Ocean colour satellites have offered sufficient spatial resolution, overpass frequency and appropriate band configuration for global-scale ocean monitoring since the launch of the ESA Medium Resolution Imaging Spectrometer (MERIS). Multispectral ocean colour sensors have been used to inform on variables such as phytoplankton pigment concentration, turbidity and transparency, suspended sediments and coloured dissolved organic matter concentrations (e.g. Binding et al., 2018; Gholizadeh et al., 2016; Odermatt et al., 2012). Ocean colour remote sensing instruments with a ground resolution in the order of 300–1000 m and high signal sensitivity have been successfully used to derive chl-*a* concentrations and turbidity in oceans with continuous global-scale observations since the launch of the SeaWIFS sensor in 1997 (Blondeau-Patissier et al., 2014; Gregg et al., 2017). They have also been used to some success to derive these parameters in medium and large inland waters (Olmanson et al., 2011; Palmer et al., 2015b; Stelzer et al., 2020). Sensors with a higher ground resolution (in the order of 10–60 m) have been available for inland applications using the Landsat series since 1972 (e.g. Carpenter and Carpenter, 1983; Guan et al., 2011; Verpoorter et al., 2014; Pickens et al., 2020). More recently, with the launch of Landsat 8 Operational Land Imager (OLI) and Sentinel-2 MSI, they have captured the interest of the research community for water quality mapping in coastal and inland waters (e.g. Pahlevan et al., 2017; Warren et al., 2019; Soomets et al., 2020; Bramich et al., 2021). MSI is of particular interest since it has wavebands centred on several of the same features as captured by OLCI and MERIS, albeit with wider bands and lower sensitivity. These common wavebands are suitable for deriving estimates of chl-*a* and turbidity, two parameters that indicate the health of the water body and potential risk factors to fish and mammals in the water.

Over the past decades, several algorithmic approaches have been suggested for deriving chl-*a* and turbidity from remote sensing data of optically complex water (for recent reviews see e.g. Odermatt et al., 2012, Matthews, 2011, Blondeau-Patissier et al., 2014). These approaches can be grouped into using analytical inversion or empirical correlation of reflectance band ratios, matrix inversion against assumed (spectral shapes of) inherent optical properties of optically active water constituents, and neural networks. Neil et al. (2019) used a global data set of in situ hyperspectral reflectance to determine that band ratio algorithms were the most robust for lakes, albeit after further optimization of each algorithm to provide the best global fit. Thus far, most algorithm development has targeted narrow (in the order of 10 nm) wavebands from recent ocean colour sensors to target diagnostic optical features. Additional effort will be needed to properly interpret the response of recent high-resolution sensors with broader wavebands. However, archives of in situ data that helped develop, calibrate, and validate biogeochemical retrieval algorithms for MERIS are still relatively scarce for more recent sensors. For inland waters, recent efforts have sought to collect together in situ observations for the purpose of satellite validation (e.g. Odermatt et al., 2018, Ross et al., 2010), similar to those attempts for the open ocean, (e.g. Werdell et al., 2003; Valente et al., 2019), but due to the relatively recent launch dates of both OLCI and MSI without dedicated global validation efforts in inland waters there are still large in situ data gaps that prevent direct calibration and validation of products derived from these sensors.

There is now greater than five years overlap between MSI and OLCI observation records, resulting in a potentially large dataset of coincident coverage. Globally validated algorithmic approaches have been used with OLCI, primarily resulting from its MERIS lineage (e.g. Smith et al., 2018; Attila et al., 2018). Some initial validation of OLCI, and expected performance based on MERIS legacy, can be found in: Stelzer et al. (2020), Soomets et al. (2020), Alikas et al. (2020a) and Liu et al. (pers. comm.). While in situ data remain scarce, we can make a direct comparison between MSI and OLCI resulting in a relative quality assessment and an opportunity to align the MSI response with OLCI. In this case, the OLCI derived chl-*a* and turbidity can be used to tune the MSI algorithms, with the benefit that these continue the MERIS legacy of in situ validation. An added advantage of this approach is that uncertainties in the retrieval of reflectance, which cannot be resolved even for MERIS due to lacking in situ radiometric data, can be largely bypassed. Nevertheless, observing inland water bodies with OLCI and other sensors is not without challenges (Palmer et al., 2015a; Giardino et al., 2019), with the most challenging issues relating to land adjacency effects (Sterckx et al., 2015, Kiselev et al., 2015), pixel resolution versus lake size (Hestir et al., 2015) and potential bottom reflectance (Lyzenga, 1981). These issues persist even in the analysis of satellite product retrieval against in situ observation data. A slight advantage of a sensor-to-sensor comparison is that much larger data volumes can be included, potentially limiting these effects which occur in specific conditions. This approach, therefore, has merit to avoid introducing bias from algorithm calibration using a more limited set of recent in situ observations. The most important question that this analysis will be able to address is whether the MSI band configuration lends itself to retrieve chl-*a* concentration and turbidity with similar performance as obtained with OLCI. This can be assessed from the distributions and statistics derived from residuals. Several algorithm-specific assumptions and observation conditions need to be taken into consideration, as discussed below.

The overall objective of this study is to establish whether, within a given set of signal processing parameters (atmospheric correction, substance retrieval algorithms), sufficient scope exists to extend the relative maturity of lake water quality observations using ocean colour sensors to smaller water bodies, using MSI and a sensor-to-sensor calibration approach. The first hurdle in this process is to address whether consistent retrieval results can be obtained at similar pixel resolutions for an optimal alignment with OLCI, within the a priori validated scope (concentration range) of each algorithm. The rationale for tuning existing algorithms is that these have already been proven to have a degree of sensitivity to the optical water properties of interest, such as chl-*a* or turbidity. They can be relied on to extract a diagnostic optical signal from satellite imagery and to model this linearly with increasing substance concentrations. However, most of the existing algorithms were originally developed without a global calibration data set, for legacy sensors, or without including atmospheric correction. All of these may result in systematic bias which can be calibrated against.

In general, it is expected that the broader MSI bands will have reduced sensitivity to the narrow absorption features of the chl*-a* pigment taken advantage of by band ratio algorithms, particularly in the red absorption band around 665 nm (31 nm width) and to a lesser extent the reference band at 709 nm (15 nm width). The less-featured NIR reflectance range used for turbidity retrieval is unlikely to show sensitivity effects caused by band width, but may still be affected by atmospheric correction uncertainties which are likely to differ between the two sensors because of differing overpass times, viewing angle ranges and the availability of bands to characterize atmospheric conditions and waterbodies. Any sensor-specific uncertainties may be amplified in the vicinity of land, as discussed further below.

For relatively clear lakes with chl-*a* in the low concentration range (in the order of 0–10 mg m^−3^), it is expected that, given adequate atmospheric correction, methods would allow the capture of chl-*a* concentration using blue-green band ratio algorithms because the MSI bands generally cover the blue pigment absorption range and the green absorption gap between prominent phytoplankton pigments. However, the wide optical diversity of lakes is likely to interfere with chl-*a* retrieval due to interference by coloured dissolved organic matter and detrital absorption in the blue, making a single algorithm calibration less likely to be globally successful. This uncertainty applies to both narrow and broadband multispectral sensors, to a likely varying and not well described extent.

Another expected challenge is the extent to which pixels at varying distance from land are affected by land reflectance mixing with water-leaving reflectance in the atmosphere. It is expected that smaller lakes and observations close to shore have a higher variance in both the chl-*a* and turbidity measurements due to varying adjacency affects in each sensor.

To draw conclusions about global algorithm performance, lakes must be selected to cover both a variety of optical water types, geographic location and altitude. We expect that lake-specific differences in algorithm performance will manifest in the algorithm response when its specificity to the target substance is relatively poor. It is then of further interest to investigate to which extent lake-specific tuning improves results locally, and whether groupings of optimized algorithm configurations can be attributed to lake size, location or optical water type.

Given uncertainties expected in the atmospheric correction procedures (Warren et al., 2019; Pahlevan et al., 2021), tuning of downstream MSI algorithms against the assumedly more capable ocean colour sensor lineage is expected to at least improve the continuity of observations between these sensors. We do not expect that specific challenges such as adjacent land effects or attribution to water types or atmospheric conditions can be fully resolved from a sensor-to-sensor comparison, because such issues will not be absent in the ocean colour sensor products. Nevertheless, our ambition for this work is to provide an immediate solution to the uptake of MSI products in a wider range of water bodies, which should then prompt more targeted validation efforts, particularly in small inland waters.

## 2 Methods

Four algorithms for chl-*a*, which performed best in the recent calibration study by Neil et al. (2019), were included: (i) the empirically derived OC2 and OC3 (O'Reilly et al., 1998) dedicated to oligo- and mesotrophic waters, (ii) a generic empirical NIR-red ratio (after Gilerson et al., 2010), which can be tuned over a large range of moderately turbid waters, and (iii) the semi-analytical Gons (Gons et al., 2002, Gons et al., 2005) algorithms. For turbidity we consider a narrower set of implementations, all adaptations of the widely validated Nechad algorithm (Nechad et al., 2010; Dogliotti et al., 2015), tuned here to use one of the four suitable MSI bands: 665, 708, 782, 865 nm for waters of increasing turbidity. To capture a diverse set of optical water types and seasonal variation, observations from 24 lakes in five continents over a two-year time frame are included in the data set.

### 2.1 Satellite data selection

**Fig. 1:**
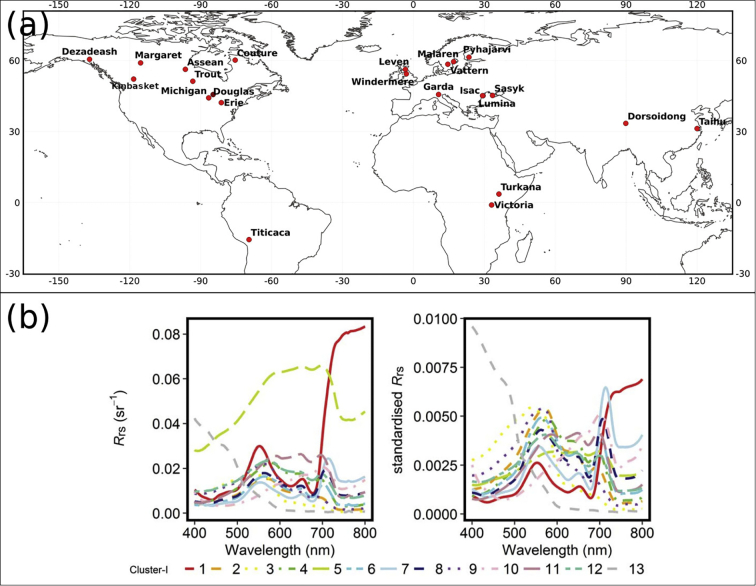
(a) Map showing lake locations used in the analysis. (b) reproduction from Spyrakos et al. (2018) showing the 13 inland optical water types.

A globally representative dataset of satellite imagery was created covering a range of lake sizes and optical water types from Europe, Africa, Asia, and North and South America (Table 1 and Fig. 1a). The surface area of the selected waterbodies ranged from 14 km^2^ to 67,000 km^2^. The selection was made to represent a variety of lake size and water types. The two-year period 2017–2018 was used to include two full seasonal cycles. It is important to include large water bodies in the analysis for reasons such as increasing the data volume for comparison (due to the coarser resolution of OLCI), to include a full variety of optical water types and for global applicability of the algorithm parameterisation. Similarly, small water bodies are included for these latter reasons and to test the limits of the experiment. In some cases, small water bodies near the boundary of the lakes chosen are included in the data set.

**Table 1:** Overview of lakes included in this study. M is the number of concurrent MSI-OLCI scenes, N is the number of concurrent MSI and OLCI pixel observations for each algorithm after filtering. Latitude and longitude given in decimal degrees. The top 3 dominant optical water types (OWT) were derived from the OLCI spectra using spectral angle classification against the OWT clusters from Spyrakos et al., 2018, with the 13 OWTs from Spyrakos et al. (2018) shown in Fig. 1b.

Lake Name (Longitude, Latitude)	Approximate Area (km^2^)	Top 3 dominant OWT	M	N OC2	N OC3	N Gilerson	N Gons
Lumina (29.5, 45.1)	13.97	3,6,9	25	80	102	53	46
Leven (−3.3, 56.3)	14.08	3,6,9	29	27	21	228	196
Windermere (−2.9, 54.3)	14.56	1,3,13	46	–	–	–	–
Isac (29.3, 45.1)	14.57	2,3,9	21	72	105	246	207
Douglas (−84.7, 45.6)	15.07	unknown	16	–	–	–	–
Dezadeash (−163.9, 60.5)	78.67	1,3,9	51	42	43	1015	680
Sasyk (33.5, 45.1)	78.96	3,5,10	16	190	179	176	176
Assean (−96.4,56.2)	79.24	2,3,4	28	180	147	1511	1418
Margaret (−115.4, 59)	80.33	1,3,9	31	371	289	70	72
Pyhäjärvi (23.5, 61.3)	120.70	2,3,4	61	455	415	2665	2444
Garda (10.6, 45.69)	368.64	1,3,13	15	13,027	13,031	–	1
Couture (−75.4, 60.1)	390.28	1,3,13	31	167	168	–	–
Dorsoidong (89.9, 33.3)	391.81	3,10,11	11	32	114	870	731
Kinbasket (−118.3,52.1)	401.09	1,3,13	35	5229	5243	22	15
Trout (−93.3, 51.1)	499.17	1,3,9	19	2739	3045	25	38
Malaren (17.1, 59.4)	1310.31	2,4,11	65	2715	1996	34,207	34,089
Vattern (14.7, 58.3)	1887.88	1,3,9	40	92,743	92,777	7713	5281
Tai (120.1, 31.1)	2416.40	4,6,11	10	1	–	686	672
Turkana (36.2, 3.5)	7566.28	2,9,12	41	31,695	585	60,974	18,782
Titicaca (−69.5, −15.8)	7752.93	1,3,9	21	91,122	91,123	36,982	31,786
Erie (−81.1, 42.1)	25,937.72	3,9,12	52	594,248	581,831	163,924	172,141
Michigan (−86.6, 44.1)	58,256.63	3,9,13	40	503,988	1,042,187	25,348	16,953
Victoria (33.1, −1.08)	67,005.94	2,3,9	89	1,353,879	945,732	357,932	353,266

Satellite images acquired by the Sentinel-2A/B MSI and Sentinel-3A OLCI were used when these were acquired within a 10-min window (using the times recorded in the image metadata). This narrow window limits the effect on data quality of different solar illumination angles and the state of the water quality between the acquisitions. However, different viewing angles and potential sun-glint effects remain. The number of concurrent satellite images ranged from 10 to 89 per lake (Table 1), depending largely on the size of the lake, satellite coverage and the temporal separation of satellite orbits. The OLCI data were from the baseline collection 002 and MSI data from processing baselines 02.04 to 02.07 depending on date of acquisition.

MSI data were resampled to 300 m pixel size to match the spatial resolution of the OLCI imagery. Following this, MSI Level-1C and OLCI Level-1B images were processed with IDEPIX v3.0 (https://www.brockmann-consult.de/portfolio/idepix/) to generate masks and then to Level 2 (normalized water-leaving reflectance, *R*_*w*_) using the POLYMER v4.12 atmospheric correction algorithm (Steinmetz et al., 2011). POLYMER was chosen as it has shown to work relatively well with MSI compared to other algorithms (Warren et al., 2019; Pereira-Sandoval et al., 2019). It is currently used in the Copernicus Land Monitoring Service for global water quality from OLCI and it has been shown to perform well in optically complex waters with OLCI (Mograne et al., 2019; Alikas et al., 2020b). The following non-default settings in POLYMER were used: no internal masking, reinitialisation of the model on a negative result, initial concentrations for chl-*a* and suspended matter set to 10 mg m^−3^ and 10 g m^−3^, respectively for the Park and Ruddick (2005) case-2 bio-optical model and the [min, max] bounds set to 0.01 and 1000 mg m^−3^ for chl-*a* and 0.001 to 1000 g m^−3^ for total suspended matter. The atmospherically corrected data were extracted and matched between the sensors on a per-pixel basis using the corresponding latitude and longitude. A 3 × 3 macro-pixel (approximately 900 × 900 m) centred on each MSI/OLCI pixel pair was then extracted to allow filtering based on spatial homogeneity (see Section 2.2).

### 2.2 Filtering observations

The following masking procedure was used to select pixels that were free from suspected effects of cloud, mixed water/land, out-of-scope use of POLYMER and high spatial heterogeneity: (1) data were masked where any of the following Idepix flags were raised on the MSI data: cloud_buffer, cloud_sure, cloud, cloud_ambiguous, cirrus_sure, cirrus_ambiguous, potential_shadow and land. In addition, each pixel had to have the Idepix MSI clear_water flag active. (2) Pixels with MSI or OLCI reflectance values >1 were removed. (3) Pixels with the POLYMER “out_of_bounds” flag for OLCI or MSI were removed from the analysis. (4) Pixels with the OLCI Idepix SNOW_ICE flag raised were also removed. (5) Following these steps, any pixel with fewer than five valid neighbours in a 3 × 3 macro-pixel was removed from the analysis.

Two additional filtering steps were added to remove outlier results and lessen the effect of edge conditions: (6) The top and bottom 0.05% of the OLCI and MSI chl-*a* distribution were removed to exclude the most extreme results, leaving the central 99.9% of data for further analyses. (7) MSI derived chl-*a* results that were greater than twice the standard deviation away from the mean of the chl-*a* dataset were removed.

Per-algorithm filtering was additionally performed to reduce the dataset to appropriate ranges of chl-*a* concentration and turbidity, as described in Table 2. These ranges are based on previous validation studies in the literature (e.g. Gons et al., 2002, Gitelson et al., 2008, Gilerson et al., 2010 and Gurlin et al., 2011 for the NIR-red ratio algorithms, and O'Reilly et al., 1998 for the blue-green ratio algorithms). The selection was based on the turbidity and chl-*a* derived from the OLCI data. No filtering was done for OCx algorithms using the MSI chl-*a* observations prior to tuning since MSI is the response variable in the tuning procedure and pre-filtering could bias the tuning result. However, some filtering by MSI chl-*a* was required for Gilerson and Gons05 to remove some data points that resulted in impossibly large chl-*a* values. OLCI-Turbidity was estimated using the Nechad algorithm (Nechad et al., 2010, Nechad et al., 2016) with the 708 nm waveband and chl-*a* was estimated using each of the four considered algorithms. It was decided to remove Lake Ladoga from all analyses after inspection showed suspect seasonal turbidity results, likely associated with (partial) ice cover. The resulting data subsets were subsequently tuned for each of the respective MSI algorithms.

**Table 2:** Algorithm-specific filtering. Observations inconsistent with these criteria were omitted from the analysis. Units of chl-*a* are mg m^−3^ and turbidity units are FNU.

	OC2 / OC3	Gilerson	Gons05	Turbidity
MSI	band ratio > 0	band ratio > 0 0 < chl-*a* < 250	0 < chl-*a* < 250	turbidity >0
OLCI	0.2 < chl-*a* < 10 Turbidity <0.5	band ratio > 0 2 < chl-*a* < 200	2 < chl-*a* < 200	turbidity >0

### 2.3 Algorithm optimization

A brief description of each algorithm configuration follows with details on their original development and validation available in the work cited below. The coefficients used for the OLCI chl-*a* algorithm configurations are the same as used for the Copernicus Land Monitoring Service (CLMS) global lake water quality processing (versions 1.2–1.4, Simis et al., 2020), except for the Gilerson and OC3 algorithms where the originally published MERIS coefficients were used (Table 3) because these algorithms are not implemented in these forms in CLMS. For MSI the coefficients used in each case are the published algorithm coefficients for MERIS as this sensor has the most similar waveband values (Table 3).

(i) OC2 and OC3 (O'Reilly et al., 1998): The ocean chlorophyll*-a* algorithms are based on the blue-green ratio and are suitable for the low chlorophyll-*a* concentration range (0–10 mg m^−3^) in waters where the pigment concentration covaries with other optically active substances. The algorithm can confuse turbid or CDOM rich waters with higher chl-*a* as these can also cause higher reflectance in the green waveband and/or decrease reflectance in the blue. OC2 uses a 490 nm: 560 nm ratio whilst the OC3 algorithm takes advantage of the 443 nm band also, using the max(443 nm, 490 nm): 560 nm ratio. These algorithms take advantage of the expected (non-linear) increase in green with respect to blue reflectance with increasing chlorophyll-*a* absorption and associated light scattering by particles. The other OCx algorithms (e.g. OC4, OC5, OC6) cannot be used with MSI as it lacks the required wavebands.
  The OC formula for chl-*a* is:
  (1) $$\log\left(\mathrm{Chla}\right)=a_{0}+a_{1}x+a_{2}x^{2}+a_{3}x^{3}+a_{4}x^{4}$$
  where
  $$\mathrm{OC}2:x=\log\frac{R_{w}490}{R_{w}560}$$
  $$\mathrm{OC}3:x=\log\frac{\max\left(R_{w}443,R_{w}490\right)}{R_{w}560}$$
  with *R*_*w*_ the normalized water-leaving reflectance and *a*_*i*_ a set of empirically tuned coefficients.
(ii) The generalised NIR-red algorithm (Gilerson et al., 2010) uses a ratio of near-infra-red and red bands (708: 665 nm) and is better suited to waters with moderate to high chl-*a* concentrations (2–200 mg m^−3^) and, because the longer wavebands show less overlap in absorption between optical substances, it is more suitable for waters where CDOM and particles do not necessarily covary with chl-*a* concentration. Around 708 nm the absorption is presumed dominated by water, whereas 665 nm corresponds to the red absorption peak of chl-*a*. The ratio of these bands is relatively insensitive to other background absorption (without spectral features in this range) and can therefore be empirically related to the chl-*a* concentration. The formula used is:
  (2) $$\mathrm{chla}={\left(\mathrm{ax}-b\right)}^{c}$$
  where $x=\frac{R_{w}\left(708\right)}{R_{w}\left(665\right)}$ and a, b, c are the coefficients subjected to calibration.
(iii) The semi-analytical Gons05 algorithm (Gons et al., 2005) uses a ratio of near-infra-red and red wavebands (708: 665 nm) together with coefficients for water absorption and chl-*a* absorption coefficients at these wavebands, and the backscattering coefficient derived from analytical inversion of reflectance at 778 nm, where water absorption is assumed to be dominant. The algorithm has previously been validated over a range of 3–185 mg m^−3^ (Gons et al., 2002) although the same band ratio has been validated in other algorithms for chl-*a* 2–200 (e.g. Gitelson et al., 2008). The included retrieval of the backscattering coefficient corrects for wider variations in the amplitude of reflectance due to detritus, minerals and phytoplankton particles (Gons, 1999).
  The formula is:
  (3) $$\mathrm{chla}=\left[\left(\frac{R_{w}709}{R_{w}665}\right)\times \left(a_{w}709+b_{b}\right)-a_{w}665-b_{b}^{p}\right]/a_{\mathrm{chl}}^{*}665$$
  where *a*_*w*_ is the absorption by pure water at the given wavelength, *b*_*b*_ the backscattering coefficient, and *p* and *a*_*chl*_^∗^are tuneable coefficients. The backscattering is calculated from:
  $$b_{b}=\frac{0.6\times a_{w}\left(779\right)\times R_{w}\left(779\right)}{0.082-0.6\times R_{w}\left(779\right)}$$

**Table 3:** Chl-*a* algorithm coefficients prior to tuning.

OLCI				MSI			
OC2 a0-a4	OC3 a0-a4	Gilerson a,b,c	Gons05 p,a*	OC2 a0-a4	OC3 a0-a4	Gilerson a,b,c	Gons05 p,a*
0.1731	0.2521	35.75	1.06	0.2389	0.2521	35.75	1.06
−3.9630	−2.2146	−19.30	0.025	−1.9369	−2.2146	−19.30	0.016
−0.5620	1.5193	1.124		1.7627	1.5193	1.124	
4.5008	−0.7702			−3.0777	−0.7702		
−3.0020	−0.4291			−0.1054	−0.4291		

All MSI chl-*a* algorithm coefficients were tuned using a non-linear least squares minimization technique (the optimize least squares package within the Python scipy library (Virtanen et al., 2020) using the trust region reflective algorithm (Branch et al., 1999)) to improve MSI – OLCI chl-*a* alignment. In the minimization procedure, a Cauchy loss function was used to reduce the effect of large outliers on the convergence, and the parameter solution was unbounded. In addition, an alternative method of tuning the OC2 algorithm has been implemented where a linear scaling of the MSI band ratio is applied to align it to the OLCI band ratio, and using the OLCI OC2 coefficients with the scaled band ratio to derive chl-*a.* This is denoted OC2scale in all relevant tables to differentiate from the traditional tuning method labelled OC2.

Four implementations of the Nechad turbidity algorithm (Nechad et al., 2010) were adapted for MSI, using the water-leaving reflectance bands at either 665, 705, 783 or 865 nm. It is a reflectance-based algorithm whose parameters are tuned based on in-water optical model and seaborne reflectance observations:

(4) $$T=\frac{A\times R_{w}}{1-\frac{R_{w}}{C}}$$

where *T* is turbidity, *R*_*w*_ is the chosen water-leaving reflectance band and parameters *A* and *C* are given as waveband-dependent look-up tables (Nechad et al., 2010; Nechad et al., 2016) and not calibrated in this study. The algorithm was linearly tuned using the formula:

(5) $$T_{t}=a*T+b$$

where *T*_*t*_ is the tuned turbidity and *a* and *b* are factors derived through the tuning. This tuning will reduce the biases originating from (a) applying the algorithm to a specific dataset of globally distributed lakes, (b) any sensor and observation differences and (c) the atmospheric correction algorithm used.

The number of observations available per lake was heavily skewed towards larger lakes (Table 1). To help mitigate this potential source of bias, each algorithm was tuned using a bootstrap analysis where each lake provided the same amount of observations, therefore giving each lake equal weighting in the minimization. Observations were selected at random, with replacement, such that each lake contributed 150 observations. Lakes with fewer than 140 unique MSI – OLCI matches were excluded. The number of observations of 150 was chosen as a compromise between the number of lakes contributing and the total number of observations in the minimization. The minimization was performed on this subset and then repeated 10,000 times. The median value of the resulting coefficients was used to calculate the optimized MSI chl-*a* and calibrated turbidity. The root mean squared difference (RMSD), mean absolute percentage difference (MAPD), mean absolute difference (MAD), bias and Pearson cross-correlation (R) (Table 4) were calculated between the OLCI and MSI derived chl-*a* (labelled before) and the OLCI and optimized MSI derived chl-*a* (labelled after) to assess the relative performance of the MSI-based retrieval.

**Table 4:** Statistical performance metrics, where *x*_*i*_ is the MSI observation and *y*_*i*_ is the OLCI observation.

MAD	MAPD	RMSD	R	Bias
$\frac{1}{N}\sum \left|x_{i}-y_{i}\right|$	$\frac{100}{N}\sum \left|\frac{x_{i}-y_{i}}{y_{i}}\right|$	$\sqrt{\frac{1}{N}\sum {\left(x_{i}-y_{i}\right)}^{2}}$	$\frac{\sum \left(x_{i}-\bar{x}\right)\left(y_{i}-\bar{y}\right)}{\sqrt{\sum {\left(x_{i}-\bar{x}\right)}^{2}\sum {\left(y_{i}-\bar{y}\right)}^{2}}}$	$\frac{1}{N}\sum x_{i}-y_{i}$

## 3 Results

From 880 concurrent MSI and OLCI observations of the selected lakes, over 17.6 million pixel matches were created and 6,703,736 pixels passed the filtering criteria. These were further filtered per individual algorithm (Table 2) to yield 2,693,002 data points for OC2, 2,779,133 for OC3, 638,972 for Gilerson and 694,633 for the Gons05 algorithm. Data from 16 lakes were used to tune OC2, OC3, Gilerson and Gons05 algorithms, out of the original set of 24 lakes (due to the bootstrapping minimum of 140 observations per lake). The greater number of matching pixels for the OC algorithms was due to relatively clear water pixels being associated with larger lakes. Some lakes, such as Lake Taihu, rarely had clear water and resulted in more observations matching the filtering criteria of the Gilerson and Gons05 algorithms. Two of the smallest lakes included in the study, Lakes Windermere and Douglas, resulted in zero matching pixels. For Lake Windermere, all concurrent MSI-OLCI observations were removed during the filtering process since all had fewer than five valid neighbours in the 3 × 3 macro-pixel, whereas Lake Douglas had no matching valid clear water after flagging and atmospheric correction.

### 3.1 Reflectance distribution

**Fig. 2:**
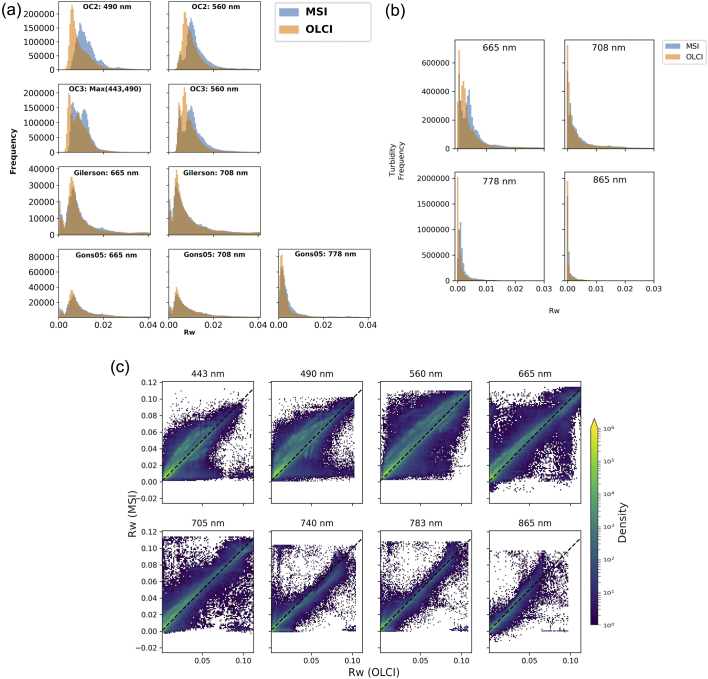
a: reflectance distributions after filtering dataset for each chl-*a* algorithm as labelled, for the bands used in that algorithm. b: reflectance distributions after filtering dataset for each wavelength as labelled, for the band used in that version of the Nechad algorithm. c: density plots (log scale) of OLCI vs MSI *R*_*w*_ for all data (unfiltered).

The distributions of water-leaving reflectance (Fig. 2a) of the OC2 dataset for 490 and 560 nm bands show an overestimation of MSI at both wavebands, with MSI showing a bi-modal distribution not seen in the OLCI data. This overestimation is also shown in the OC3 max(443,490) and 560 comparison, but both sensors show a bi-modal distribution. The 665 and 708 reflectance in both Gilerson and Gons05 show much more aligned histograms, with the bi-modal distribution matching well between both sensors, and the Gons05 778 nm aligns well too. The distribution of reflectance for the turbidity dataset (Fig. 2b) shows an overestimation in MSI *R*_*w*_ of the second peak, most prominent in the 665 nm band, and for higher wavelengths much reduced signal. Density plots of the *R*_*w*_ without the limits from Table 2 applied (Fig. 2c), for each band, show a high density along the line of unity but with prominent scatter, particularly in the 443, 490 and 560 nm bands.

### 3.2 Adjacency effects

**Fig. 3:**
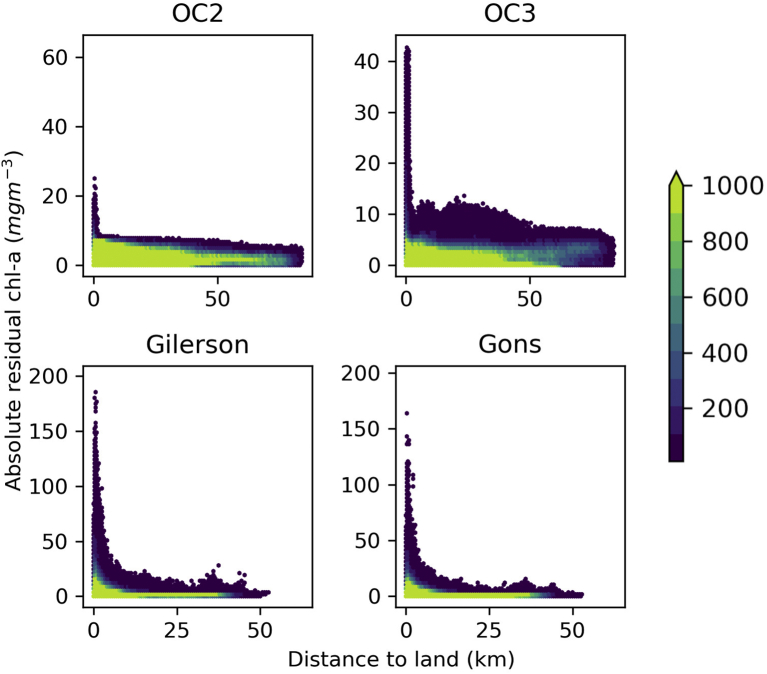
Density plots showing residual chl-*a* versus distance to land for the four chl-*a* algorithms. The colour denotes the number of observations per cell.

The difference of MSI and OLCI chl-*a* was compared to the distance to the nearest land map from Carrea et al. (2015). There was a clear increase in the magnitude of residual error at distances close to land (Fig. 3) for each of the algorithms. The effect, in terms of absolute difference between MSI and OLCI chl-*a* retrieval, was stronger and visible at greater distance from land for the red band ratio algorithms compared to the OC2 algorithm. Overall, the number of observations with elevated residuals was far fewer than those with small residuals, even at the distances close to land. For example, at distances <5 km for OC2, 97.5% of the data had residual less than 7.27 mg m-3 (the maximum error at 80 km distance to land). Statistical analyses were performed using a dataset of points < 5 km and another >5 km from land described in Table A in Appendix A. From these statistics, it was decided to keep all observations in the dataset in further analyses rather than restrict the tuning to only the larger lakes, which would then reduce the dataset and potentially narrow the range of optical properties and global applicability.

### 3.3 Chl-*a* algorithm optimization

**Fig. 4:**
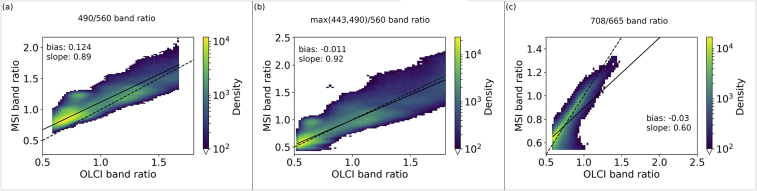
Band ratios for the (a) 490: 560 nm (b) max(443,490): 560 nm and (c) 708: 665 nm wavebands. Note that pixels with density of fewer than 100 are not shown. The dotted line shows unity and the solid line regression.

Statistics relating the OLCI and MSI chl-*a* were produced both before (Table 5) and after (Table 6) algorithm optimization. As well as looking at the statistics it is prudent to investigate the intermediary products. The water-leaving reflectance band ratios derived from OLCI and MSI can be seen to be strongly correlated (Fig. 4). The correlation suggests that the algorithms can be applied to MSI, particularly with further tuning of MSI-specific algorithm coefficients. Considering OC2, the 490: 560 nm band ratios, the MSI is generally over-estimated compared to the OLCI (Fig. 4a) with a bias of 0.124 and regression slope of 0.89. The max(443,490): 560 ratio appears more centred with bias −0.011 and regression slope 0.92 (Fig. 4b). The 708: 665 ratio shows a general trend of underestimation by MSI with bias −0.03 and regression slope 0.60 (Fig. 4c).

**Table 5:** Descriptive statistics of MSI vs OLCI derived chl-*a* before optimization.

	MAD	RMSD	BIAS	R	MAPD
OC2	1.95	2.67	−1.54	0.61	75.9
OC3	1.18	2.34	−0.04	0.74	37.62
Gilerson	4.68	11.27	−1.79	0.67	41.37
Gons	3.73	8.79	−0.91	0.67	37.7

**Table 6:** Descriptive statistics of MSI vs OLCI derived chl-*a* after optimization.

	MAD	RMSD	BIAS	R	MAPD
OC2	1.11	1.61	−0.40	0.80	54.47
OC3	1.16	1.65	−0.51	0.88	45.68
Gilerson	4.64	11.14	−1.54	0.68	44.52
Gons	3.67	8.92	−2.5	0.67	37.07
OC2scale	1.36	2.23	−0.29	0.72	51.77

**Fig. 5:**
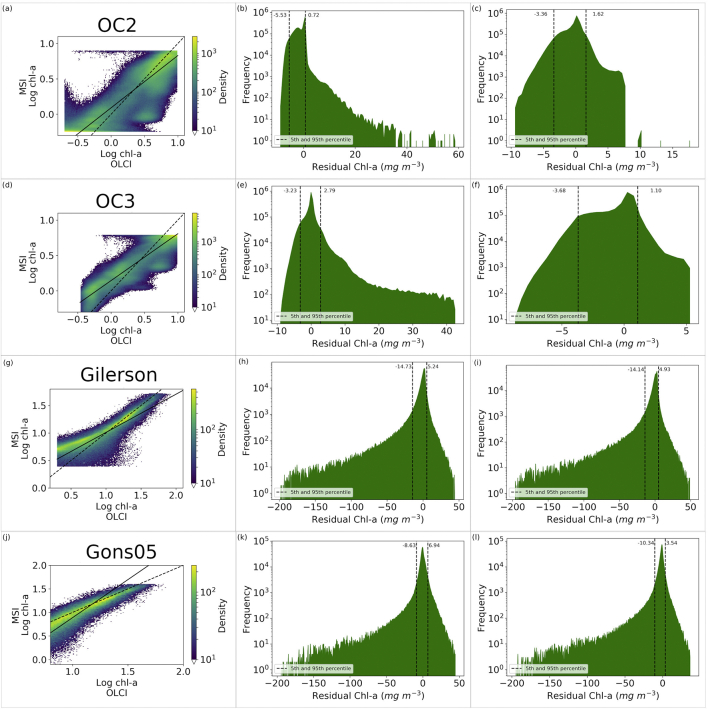
Panel of results showing (column 1) optimized log chl*-a* plots, (column 2) residuals prior to optimisation and (column 3) residuals after optimisation. Each row is for a different algorithm (OC2, OC3, Gilerson and Gons05 from top to bottom). Dashed lines on residual plots show the 5th and 95th percentiles. Solid and dashed lines on the density plots show the regression and line of unity respectively.

MSI derived chl-*a* using the initial algorithm coefficients for OC2 is generally overestimated for log chl-*a* < 0 and underestimated for log chl-*a* > 0, compared to OLCI derived chl-*a*, with clustering of results around 0.5 and 4.0 mg m^−3^ and relatively poor agreement from the bias −0.11 mg m^−3^ and regression slope of 0.34. Optimization of the algorithm coefficients successfully removed most of the bias (resulting bias 0.01 and regression slope 0.74), while a clustering of results remained evident (Fig. 5a). After optimization 90% of the residuals lie between −3.36 and 1.62 (Fig. 5c). The bootstrap analysis of the coefficients showed that the higher power terms were subject to wider variation than the lower power terms (Appendix C: Table C1), with an interquartile range of 30 for the a_4_ term (−31.5261) compared to 0.01 for the a_0_ term (0.3818), suggesting a high dependency on the input dataset.

For the OC3 algorithm, the middle 90% spread of the optimized residuals were between −3.68 and 1.10 (Fig. 5f). The regression slope and bias are better before (0.96 and − 0.01) than after (0.66 and − 0.03) optimization. The interquartile ranges of the bootstrapped coefficients were markedly narrower than for the OC2 optimization (in particular for coefficients a_2_, a_3_ and a_4_ – Appendix C), suggesting that the optimization of OC3 was not as sensitive to the input data set as observed with OC2.

The results for the Gilerson algorithm optimization show the middle 90% of the residuals range from −14.09 to 4.92 mg m^−3^ (Fig. 5i) with a frequency peak close to 0 mg m^−3^. The bias decreased from −1.79 to −1.54 mg m^−3^ and the RMSD decreased by 0.13 mg m^−3^, however the MAPD increased by 3.2%. The optimized log chl-*a* (Fig. 5g) shows good agreement along the axis of unity for values of log chl-*a* > 1, but the MSI derived chl-*a* is generally overestimated for log chl-*a* < 1. The interquartile ranges of the bootstrapped coefficients (Appendix C) were 1.5, 0.9 and 0.1 for a (9.38), b (−3.38) and c (1.73) respectively.

After optimization the Gons algorithm middle 90% of residuals (Fig. 5l) were between −10.30 mg m^−3^ and 3.52 mg m^−3^ with the peak close to 0. The optimized chl-*a* (Fig. 5j) shows an alignment along the axis of unity, with a small reduction in MAPD (0.6%) and MAD (0.06 mg m^−3^) but an increase in bias (−1.5 mg m^−3^) and RMSD (0.14 mg m^−3^). The interquartile range of the bootstrapped coefficients were tight, with 0.01 for p and 0.0003 for *a*_*chl*_^∗^.

The optimized coefficients for each algorithm (Table 8) differ from the initial substantially in most cases. The deviation is shown as a multiplier of the initial coefficient values. It can be seen that the Gons05 tuned coefficients are most similar to the initial coefficients, with p increasing by 0.2% and *a*_*chl*_^∗^ by 20%. A reduction of the Gilerson coefficients a and b is seen, compared to an increase for c. For the OC2 and OC3 algorithms, most coefficients increase in magnitude, also with a change in sign for a_2_ and a_3_ for OC2 and a_3_ for OC3.

#### 3.3.1 Per-lake optimization

**Fig. 6:**
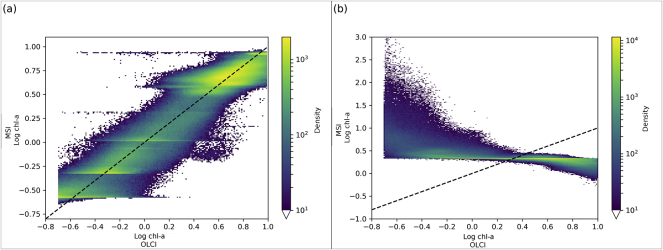
(a) Density plot of per-lake optimized log chl-*a* using 16 different parametrizations – one for each lake. (b) Density plot of optimized log chl-*a* using the median of the per-lake coefficients.

The clustering of points evident in Fig. 5a further suggests that algorithm performance varies between (groups of) lakes. Lake-wise optimization of the coefficients can express the difference in algorithm performance between the global algorithm solution and locally optimized algorithms. To investigate the sensitivity of the algorithm tuning to local variation, the algorithm coefficients were also optimized on a per-lake basis and compared with the combined solution above. Note that bootstrapping was not used in the per-lake optimization since there was no need to remove bias caused by larger lakes. Statistics describing the per-lake tuning are in Appendix B (Table B1) together with derived OC2 coefficients (Table B2). The per-lake solutions showed wide variation (Table 7) and hence a strong dependence on the tuning dataset. As an example, a sample which yielded a chl-*a* concentration of 2.0 mg m^−3^ using the bootstrapped solution yielded 7.7 mg m^−3^ with the equivalent algorithm based on the median coefficients of the per-lake solutions. When combined, the per-lake optimized data (Fig. 6a) show correlation along the axis of unity, but hide the poor performance of some lakes, which may be seen as horizontal strips with little correlation between OLCI and MSI (e.g. at around y = 0.9, y = 0.3, y = 0.02). The data after optimizing using the single set of the median parameters shows little correlation along the axis of unity (Fig. 6b), simultaneously showing the merit of the bootstrapped analysis to arrive at a global solution, and potential limitations of the OC2 algorithm specificity.

**Table 7:** Statistics describing distribution of OC2 coefficients (a_0_ – a_4_) optimized per lake.

Coefficient	Mean	Standard dev.	Min	Median	Max
a_0_	0.97	3.5	−6.5	0.3	12.2
a_1_	36.05	99.9	−33.1	−0.1	392.7
a_2_	186.58	1156.7	−2045.8	−2.6	4647.3
a_3_	1806.94	5615.6	−4658.2	85.5	23,992.5
a_4_	2363.63	12,951.5	−18,871.9	80.6	45,903.6

**Table 8:** Chl-*a* algorithm coefficients after optimization and their deviation from the coefficients prior to tuning, as a multiplier of their initial values.

Optimized MSI				Deviation of coefficients (multiplier)			
OC2 a0-a4	OC3 a0-a4	Gilerson a,b,c	Gons05 p,*a*_*chl*_^∗^	OC2 a0-a4	OC3 a0-a4	Gilerson a,b,c	Gons05 p,*a*_*chl*_^∗^
0.3818	0.3121	9.3803	1.0624	1.60	1.24	0.26	1.002
−4.9640	−1.7612	−3.3763	0.0192	2.56	0.79	0.17	1.2
−0.9966	2.9117	1.7304		−0.57	1.92	1.54	
57.3857	3.2944			−18.6	−4.28		
−31.5261	−28.3593			299.1	66.1

**Table 9:** Statistics describing the fit of OLCI and MSI derived turbidity for wavelengths 665, 708, 783, 865 nm before optimization. RP_n_ denotes the n^th^ percentile of the residuals.

wavelength	MAD	RMSD	BIAS	R	MAPD	RP_5_	RP_50_	RP_95_
665	0.85	2.28	0.54	0.96	147.6	−3.3	−0.32	0.14
708	1.22	4.91	0.69	0.91	435.17	−4.12	−0.27	0.33
778	1.93	12.13	1.12	0.76	702.28	−4.07	−0.91	0.04
865	1.52	18.06	−0.36	0.66	641.68	−1.20	−0.24	2.33

**Table 10:** Statistics describing the fit of OLCI and MSI derived turbidity for wavelengths 665, 708, 783, 865 nm after optimization. RP_n_ denotes the n^th^ percentile of the residuals.

wavelength	MAD	RMSD	BIAS	R	MAPD	RP_5_	RP_50_	RP_95_
665	0.72	2.43	0.10	0.96	115.18	−1.84	−0.15	0.71
708	1.10	4.80	0.16	0.91	460.22	−2.66	−0.17	0.95
778	1.55	11.55	−0.04	0.76	371.69	−2.19	−0.23	1.62
865	1.52	18.05	−0.41	0.66	626.34	−1.16	−0.22	2.48

**Table 11:** MSI algorithm tuning coefficients for the Nechad et al. (2010) turbidity algorithm using four different MSI wavebands.

	665 nm	708 nm	778 nm	865 nm
a	0.882	0.868	0.843	0.990
b	−0.024	0.087	−0.333	−0.008

**Fig. 7:**
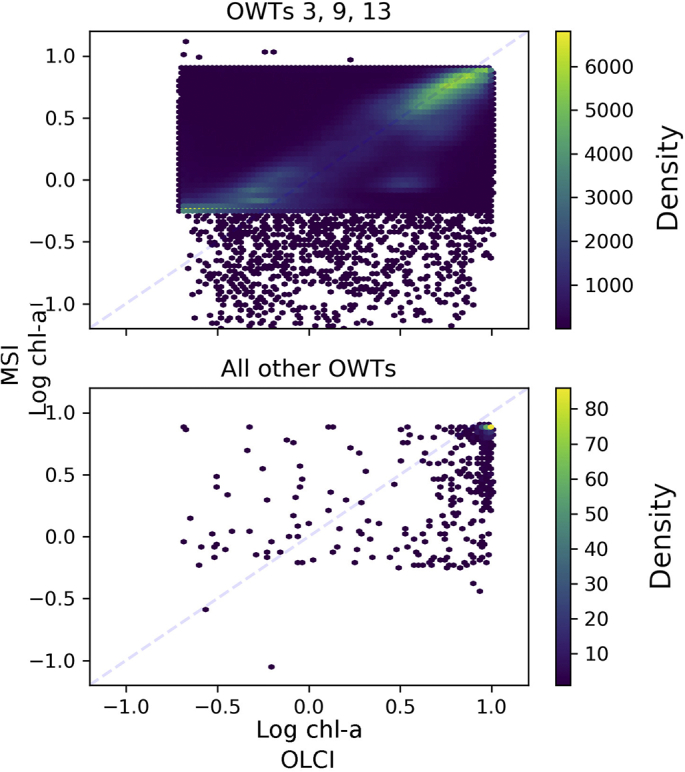
Dominant optical water type clustering of OC2 derived chl-*a* observations. Shading denotes the density of points per-pixel.

To investigate the apparent clustering further, we determined the similarity of the OLCI spectra to the inland optical water types (OWT) developed by Spyrakos et al. (2018). Note that matching MSI data to MERIS/OLCI derived OWTs is not ideal since the sensors are optically different (e.g. configuration of wavebands) and therefore detect different features. To date there is not a set of global OWTs derived from MSI data available, however, using OLCI derived OWTs as reference allows an initial investigation. The dominant OWT was identified for each OLCI spectra and used to separate algorithm results per OWT. The MSI-tuned OC2 algorithm consistency was inspected against OLCI-derived chl-*a* (also using OC2). As expected from the a priori filtering, most of the data where the OC2 algorithm was applied correspond to types 3, 9 and 13 (Fig. 7), which are the OWTs with deepest secchi depth. After algorithm optimization, consistency between the two sensors is highest in these OWTs, whereas the data points from the other OWTs have larger variability. This can be described by the following statistics: *R* = 0.8, bias = −0.4, MAD = 1.11, MAPD = 54.5 and RMSD = 1.6 for the set of OWTs 3, 9 and 13. For the set of all other OWTs the corresponding statistics are: *R* = 0.57, bias = −2.6, MAD = 2.98, MAPD = 62.7 and RMSD = 3.57.

#### 3.3.2 Linear scaling

**Fig. 8:**
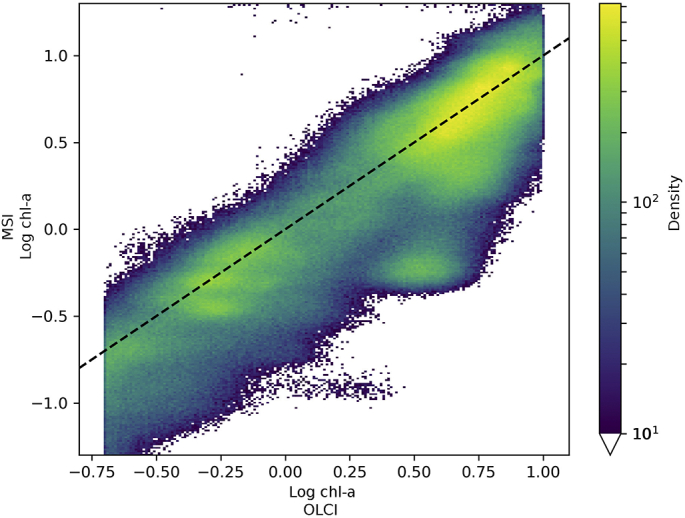
Log chl-*a* plot of the OC2 algorithm when using the scaled MSI band ratio with OLCI coefficients (OC2scale). Dashed line is the line of unity.

The relationship between MSI and OLCI band ratios (Fig. 4a) appears approximately linear over the range of data, which suggests that, as an alternative approach to tuning the algorithm coefficients, for those methods only dependent on the band ratio, a linear correction of this band ratio could suffice to align the MSI response with OLCI. To test this, for OC2, the linear function was derived as:

(6) y=1.442x−0.51

where *x* is the MSI band ratio. Using the resulting scaled band ratio along with OLCI coefficients the OC2 chl-*a* was derived (Fig. 8). This aligns well with the axis of unity and shows a reduction in the bias of 1.2 mg m^−3^, RMSD of 0.4 mg m^−3^ and MAPD of 24% and has 90% of residuals between −3.60 and 2.84 mg m^−3^.

#### 3.3.3 Spatial analysis

**Fig. 9:**
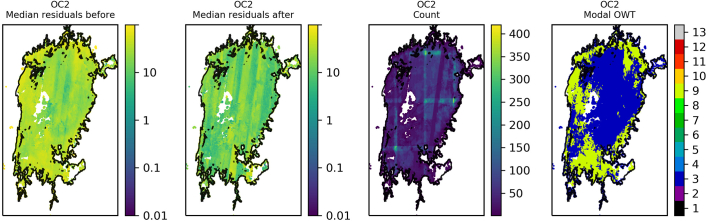
Spatial overview of the median residual (a) before and (b) after optimisation of the OC2 algorithm, (c) the number of observations and (d) the modal dominant optical water type per pixel for Lake Victoria.

**Fig. 10:**
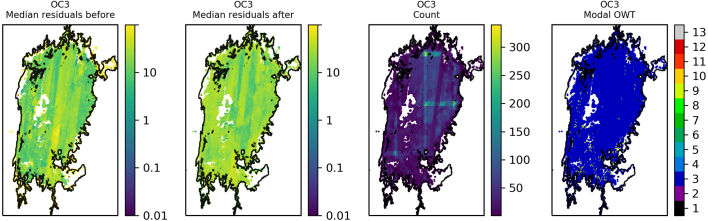
Spatial overview of the median residual (a) before and (b) after optimisation of the OC3 algorithm, (c) the number of observations and (d) the modal dominant optical water type per pixel for Lake Victoria.

**Fig. 11:**
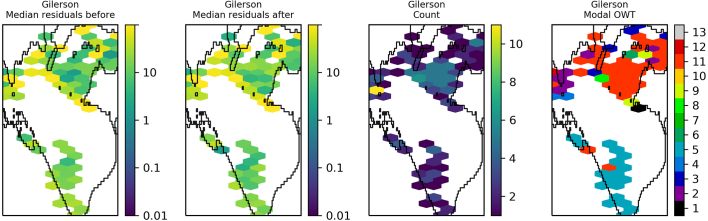
Spatial overview of the median residual (a) before and (b) after optimisation of the Gilerson algorithm, (c) the number of observations and (d) the modal dominant optical water type per pixel for Lake Sasyk.

**Fig. 12:**
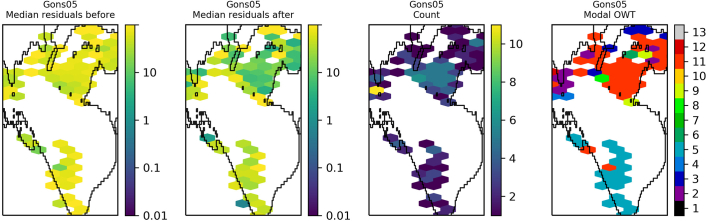
Spatial overview of the median residual (a) before and (b) after optimisation of the Gons05 algorithm, (c) the number of observations and (d) the modal dominant optical water type per pixel for Lake Sasyk.

The residuals were binned and plotted per lake to identify spatial patterns in the data. Lake Victoria (Fig. 9) shows a general reduction in median residual after optimisation of OC2, although striping of MSI detectors is clearly visible in the data after optimisation. The same for OC3 (Fig. 10) shows median residuals in certain areas of the lake were increased but also that there was a decrease of the largest median values (e.g. central region and north east). Lake Sasyk (Fig. 11, Fig. 12) qualitatively shows little change after optimisation of the Gilerson algorithm whereas with the Gons05 optimisation there is a clear reduction in median residual across the lake.

### 3.4 Turbidity

**Fig. 13:**
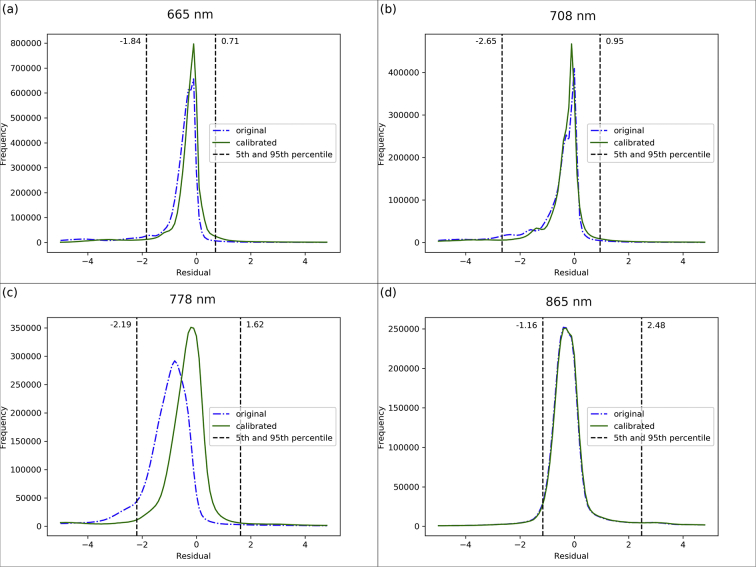
(a) Distribution of residual turbidity values calculated from 665, 708, 778 and 865 nm bands.

There were, after filtering, 5,119,210 observations used in the turbidity algorithm calibration for the algorithm based on the 665 nm band, 3,183,299 for the 708 nm band algorithm, 4,790,170 for the 778 nm band and 2,977,693 for the 865 nm band. The bootstrapping incorporated observations from 20 lakes in the 665 and 708 nm calibration, 21 in the 778 nm and 13 lakes in the 865 nm calibration. The residual distributions before and after calibration (Fig. 13) show that the residuals after tuning gave a median closer to 0 and a tighter interquartile range except for the 865 nm case which showed a relatively small difference. Not shown is that the turbidity data were in general skewed to lower values, with approximately 80% of the dataset at <5 FNU. For the wavebands 665, 708 and 778 nm, the bias and MAD all reduced after optimization (Table 9, Table 10). The RMSD increased for the 665 nm waveband but decreased for the 708 and 778 nm wavebands, and the MAPD decreased for 665 and 778 nm but increased for 708 nm. There was little change for the 865 nm waveband. The gain values, *a*, (Table 11) are below 1.0 for each waveband suggesting MSI typically overestimates turbidity compared to OLCI prior to calibration. Welch's *t*-test was performed for each waveband and in all cases the null hypothesis that the calibrated turbidity sample mean equalled the initial turbidity sample mean was rejected.

## 4 Discussion

Despite offering broader wavebands, the MSI detector shows distinct capability to adopt established algorithm concepts for the retrieval of chl-*a* as supported by the statistics in Table 5, Table 6. In general, as expected, the retrieval precision of the MSI algorithms appears lower in comparison with OLCI, resulting in wider distributions across the range of observed chl-*a* concentrations. This alignment of MSI algorithms with OLCI demonstrates four potential advances for remote sensing of lakes at high resolution:

- A more consistent retrieval of chl-*a* and turbidity from lakes across multiple sensors
- An increase in the confidence of observations for smaller lakes, albeit with caution near adjacent land
- possible future integration of data and increased number of observations by combining OLCI and MSI
- Resolving refined spatial structures in chl-*a* and turbidity within lakes observable by OLCI

However, there are implications of using a sensor-to-sensor tuning. The residual error from atmospheric correction influences the tuning, therefore the tuned algorithms will perform best with data corrected by the same correction algorithm. Similarly, if either the level1 data are reprocessed resulting in different radiances, or upgrades to the atmospheric correction algorithm are released, then the tuning would likely also need to be repeated.

Relating the residual chl-*a* to distance from land (Fig. 3) showed that the largest deviations of MSI derived results from OLCI tend to be closer to land, which suggests that the adjacency effect had a differential effect on MSI and OLCI retrieval. It is not possible to determine which observation approach is more affected, or whether either results in acceptable product uncertainty, but this confirms that product uncertainty is relatively high in these environments. Thus, combining the two sensor types to derive a smooth transition from nearshore waters at high resolution, while adopting the more sensitive medium-resolution sensor results in open water, may not be straightforward. The largest deviations appeared to be restricted to distances less than 5–10 km to the shoreline, while there were also many observations with small deviations within this distance range. Appendix A showed tuning with match-ups greater than 5 km distance to shore didn't make a large difference statistically to using the whole data set, but had the penalty of removing the smaller lakes from the data set. It would be of interest to further explore whether the deviations are associated with optical properties of the atmosphere, water or surrounding land at the time of observation. Bulgarelli and Zibordi (2018) showed that adjacency effects can exceed radiometric noise thresholds at distances from land of 36 km for OLCI and 20 km for MSI when the land surface is bright (e.g. sand, snow), and around 10 km for MSI for darker reflecting surfaces. Residual error distributions did not show any clear trends when plotted as per-lake or by latitude (results not shown).The distributions of POLYMER-derived *R*_*w*_ for OLCI and MSI were comparable, especially for the 665, 708 and 778 nm bands used in the chl-*a* tuning (Fig. 2a). This suggests the atmospheric correction was consistent between the sensors in the red to NIR spectral region, which is comforting because chl-*a* and turbidity retrieval from these bands on ocean colour sensors have already been more widely validated for the >10 mg m^−3^ concentration range (e.g. Odermatt et al., 2012). Considering the distributions in the turbidity dataset (Fig. 2b), which had less restrictive filtering than the chl-*a* dataset, there may be an over-estimation in MSI compared to OLCI in the 665 nm waveband. The 490 nm and 560 nm bands showed an elevated atmospherically corrected reflectance for MSI compared to OLCI, which suggests the atmospheric correction does not perform consistently in these wavebands. This can also be seen in the reflectance scatter plot (Fig. 2c) with the 443 to 665 nm wavebands showing many pixels with elevated Rw for MSI, but also more pixels on the one-to-one line. This inconsistency can be explained when considering the POLYMER atmospheric correction relies on populating a bio-optical model with realistic concentrations of optically active water constituents. With optically complex waters, water itself is the only consistent strong absorber and this is mostly visible in the red, NIR and short wave infra-red (SWIR). Thus, the uncertainty of atmospheric correction increases towards shorter wavebands. When a sensor provides more narrow bands, finding the optimum solution of the various atmospheric and water components benefits from more degrees of freedom. MSI has a limited set of visible and NIR wavebands (8 in the range 443–865 nm vs 15 for OLCI). The OLCI sensor follows on from MERIS, as does its validation through match-up analyses (Attila et al., 2018, Alikas et al., 2020a, Stelzer et al., 2020), and together these explain why, at least in theory, the OLCI product should be the reference.

The OC algorithms using a global parameterization show effects visible as clustering of points (e.g. Figs. 4a, b, 5a, d). Although some filtering was performed to remove points with turbidity >0.5 FNU and chl-*a* > 10 mg m^−3^, other optical scattering substances or CDOM could remain and make the use of (globally tuned) OC algorithms a poorly performing solution in those waters. Bottom reflectance is a particular risk in clear waters and can in part be accounted for by the filtering applied, while some of the MSI pixels may still show these effects due to a higher spatial resolution. Bio-optical diversity in inland waters is very wide and can be studied in terms of phytoplankton community composition, phenotypic variability resulting in local or seasonal differences in e.g. pigment expression or cell size in the phytoplankton community. These effects are governed by environmental conditions including seasonality, nutrient load, vertical mixing and weather patterns. Algorithms targeting the absorption features of chl-*a* are subject to uncertainty due to this natural variability and physical forcing. Therefore, local optimization of non-analytical retrieval algorithms is always likely to outperform any global solution. It is, however, only advisable to follow local tuning if validation can be carried out systematically, to avoid biases in the calibration data set.

Per-lake tuning of the chl-*a* algorithms (Table 7) shows a wide variation in the optimized parameters of the OC algorithms for MSI. For example, the standard deviation for coefficients a_2_, a_3_ and a_4_ was greater than 1000 with min-max ranges in the thousands, suggesting that the OC algorithms cannot be parameterized in a global fashion and per-lake or per optical water type parameterization would perform better. There is also wide variation between those coefficients for lakes with similar optical water types, such as lakes Garda, Couture and Kinbasket. This could result from localised differences including adjacency, atmospheric correction performance and a large variation in number of observations, all of which can be influenced by lake size and shape, surrounding environment and altitude. The variable presence of CDOM in different lakes could largely affect the *R*_*w*_ in blue bands, and therefore the OCx algorithm coefficients within the tuning. Appendix B lists the coefficients derived for each individual lake. The per-lake optimized data (Fig. 6a) showed good correlation along the axis of unity, which may be expected since the algorithm is tuned specifically for each lake. Nevertheless, using the new global algorithm parameterization, the distribution of MSI chl-*a* (Fig. 5a) was still closer to that of OLCI than the MERIS parameterization of MSI chl-*a.* This is reflected in the residual plot (Fig. 5c) which shows a peak centered close to 0 and that 90% of residuals lie between −3.3 and 1.6 mg m^−3^.

Separating the algorithm configuration by (global) optical water types would provide an intermediate solution between a single global optimization such as presented here, and tuning of algorithms per lake or location, which is impossible to achieve for the vast majority of lakes. Thus far, OWT characterization with MSI is still limited (e.g. Soomets et al., 2020) and should be further explored at the global scale. The limited bandset (fewer and wider wavebands) of the MSI instrument do not allow all 13 optical classes of Spyrakos et al. (2018) to be distinguished from MSI data. From corresponding OLCI spectra it was shown (Fig. 7) that the optical water types 3, 9 and 13 (those described with highest transparency) consist of the majority of data points and that these lie approximately on a straight line after optimization. Data points from the other OWTs were scattered, and therefore are likely unsuitable OWTs for the OC2 chl-*a* algorithm. For future work, a global set of MSI OWTs needs to be developed to allow filtering and algorithm selection based on the MSI data itself, rather than OLCI, which should reduce some of the uncertainty seen in the results.

An alternative approach to the OC2 algorithm tuning was used where the MSI band ratio was mapped, using a linear function, to the OLCI band ratio range, and the OLCI OC2 coefficients used to derive chl-*a*. This is possible because the two sensor ratios showed a linear relationship (Fig. 4a) and a sensor-to-sensor tuning is being followed. Statistically, the RMSD and MAD are higher, the bias, R and the MAPD are lower for the chl-*a* derived here compared to the traditionally tuned algorithm (Table 6). The chl-*a* derived via this method does not show the same clustering at the lower limit as seen in the traditional tuning method (Figs. 5a, 8). The highest order coefficient (a_4_) shows a change in magnitude of 299 times in the traditionally tuned method, which may suggest the algorithm's relationship to the (bio)physics, in this tuned form, has been weakened, and is likely why there is a large cluster of points at the lower limits in Fig. 5a. It is noted that the OC3 result does not show this cluster of points at the lower limits. As a similar result can be attained by tuning the band ratio and using coefficients that work for OLCI, it is important that future work concentrates on the reliability of the MSI band ratio (e.g. atmospheric correction) as this is the limiting factor to deriving chl*-a* with this algorithm*.* The Gilerson and Gons05 NIR: red band ratios (Fig. 4c) showed a tight agreement between the OLCI and MSI reflectance distributions and improvement in the optimized chl-*a* distributions, providing confidence that consistent atmospheric correction and constituent concentration retrieval is feasible between these sensors. The optimized Gilerson MSI chl-*a* (Fig. 5g) agrees well with the OLCI log chl-*a* for values >1 but shows general overestimation for log chl-*a* values <1, hence may not be applicable in waters with chl-*a* < 10 mg m^−3^. Optimization of the Gons05 MSI chl-*a* (Fig. 5j) has resulted in a chl-*a* distribution whose centre of gravity is more along the line of unity. Residual plots for both Gilerson and Gons05 (Fig. 5i,l) show tails deeper into negative residual (i.e. MSI underestimation) than the OCx, but the frequency count is low (e.g. for Gilerson there is <0.01% of dataset with residual < −50 mg m^−3^). The mid 90 percentile ranges after optimisation are reduced in all cases suggesting an improved global tuning.

For the OC2 algorithm, the MAD, RMSD, bias and MAPD showed improved performance after algorithm coefficient optimization and R increased from 0.61 to 0.80. The OC3 algorithm showed improvement such as a decrease in RMSD from 2.34 mg m^−3^ to 1.65 mg m^−3^ and increase in R from 0.74 to 0.88. For the Gilerson and Gons05 algorithms the improvements in the overall statistics were far more subtle (Table 4, Table 5). Of course, these algorithms have both different underlying theory and use cases (OC algorithms for oligo-mesotrophic waters, Gilerson and Gons05 for more turbid waters). The improved alignment of MSI-OC2 and OC3 algorithms with OLCI retrieval suggest that algorithms that use the blue and green MSI bands will benefit most from the configurations derived here. The first reason for this result is perhaps that there was no established MSI configuration for the OC algorithms, and the ‘initial’ reference here used a configuration that was tuned for MERIS retrieval. Moreover, reflectance in the blue and green bands differed more with respect to OLCI than the red and NIR bands (Fig. 2a). Despite red and NIR reflectance from MSI, corrected for the atmosphere using POLYMER, showing good consistency with OLCI, previous studies have shown these bands, after atmospheric correction, are more uncertain than the blue and green (Warren et al., 2019; Pahlevan et al., 2021). If we (realistically) assume the retrieval of reflectance from OLCI to be relatively more reliable, these results provide another indication that atmospheric correction of MSI over water should remain a research priority.

Although the distance to land experiment shows that the highest residuals are from matches close to land, there are still unexplained biases present in the data set. No obvious patterns in the residuals emerged when plotted versus lake, latitude or OLCI optical water type. Atmospheric correction performance will vary per scene due to an array of factors discussed in the literature (e.g. uncertainties in models, aerosols, illumination conditions) and is one likely source of residual error. Another possible source could be low signal sensitivity in the MSI over clear water or additional signal in glint affected waters. Future work should consider identifying these regions to investigate the suitability of MSI water quality in such affected waters.

In this work we compared two chl-*a* algorithm strategies, those based on blue-green and NIR-red band ratios, as these have proven effective over the low and medium-high chl-*a* concentration range, respectively. Alternative methods, ranging from analytical to empirical, and the use of Optical Water Types (e.g. Moore et al., 2014) to deploy appropriate algorithms, or machine learning techniques (e.g. Pahlevan et al., 2020) to extend the range of applicability of more complex statistical solutions, can of course also be considered. However, the limitations of MSI in reproducing the chl-*a* retrieval of dedicated ocean colour sensors can be established from the bands that carry the most important optical information for characterizing phytoplankton presence.

Turbidity was calculated using coefficients from the published look-up tables in Nechad et al., 2010, Nechad et al., 2016, which were generated for use with narrow band sensors. MSI does not have such narrow red/NIR bands (bandwidths ranging from 15 nm to 31 nm), and while the NIR bands do not typically show narrow reflectance features, these algorithms may need further tuning to align the MSI and OLCI responses. Coefficients for MSI are available in the ACOLITE software package but have not been used here because they needed to be separately assessed for POLYMER. This can be achieved in part by applying a linear scaling function to the turbidity after calculation, as performed here. The MAD and bias after such optimization (Table 10) showed improvements for all wavebands except at 865 nm. The MAPD decreased for 665, 778 and 865 wavebands whereas the RMSD showed a slight increase at 665 and 708 nm and a slight improvement for the other wavebands. The 95-percentile range of the residuals decreased after calibration for all but the 865 nm waveband and for all wavebands the median residual value is closer to 0 after optimization. With increasing wavelength, we can observe a tendency for the RMSD to increase and R to decrease. This is likely a reflection of predominantly low to medium-turbidity waters in the dataset, as longer wavebands are more appropriate for increasingly turbid waters. This is backed up by the reflectance distributions (Fig. 2b) showing large number of observations with very low *R*_*w*_ for increasing wavelength, and almost twice the number of observations at 665 nm than at 865 nm. It is suspected that the coefficient *a* for waveband 865 is close to 1 due to the low reflectance at this wavelength. Investigating particularly turbid waters in future, such as estuaries, may add further information suitable for this waveband.

An integral part to adopting a global MSI approach using these algorithms is the availability of OWTs derived from MSI itself, to enable an OWT switching approach, and the mapping of algorithms to the OWTs. An optical water switching approach for MSI data would require the following work undertaken in future:

• Creation of a MSI derived set of global optical water types with assignment to chl-*a* and turbidity algorithms.
• Application of these tuned algorithms to water bodies of varying size, shape, altitude, latitude etc. in conjunction with organised validation campaigns to establish how uncertainties vary with observation conditions and spatial resolutions.

## 5 Conclusions

A data set of near-coincident MSI and OLCI observations over inland waters was used to (further) tune chl-*a* and calibrate turbidity algorithms from previously published formulations. Four algorithms for chl-*a* (OC2, OC3, Gilerson and Gons05) and a turbidity algorithm (Nechad) were used and coefficients derived to improve the algorithm performance with MSI. All algorithms show statistical improvement after optimization, with blue-green chl-*a* ratio algorithms benefiting most from the proposed adjustments. These results show that MSI observations can be used to complement chl-*a* and turbidity obtained from OLCI. However, significant uncertainty remains, particularly near land as a result of adjacency and/or shallow water effects. There is further uncertainty related to the optimal application domain of each algorithm, particularly at low chl-*a* concentrations (< 10 mg m^−3^) where blue - green band ratio algorithms are sensitive to the presence of other optically active substances, thus requiring additional information to adopt the most suitable algorithm for each condition.

With monitoring regulations such as the European Water Framework Directive and ambitious objectives for the quality of ambient water under UN SDG 6.3 it is of increasing importance to be able to estimate lake water quality parameters such as chl-*a* and turbidity in an efficient manner. For all countries to be able to report these figures for all relevant water bodies the use of Earth observation is vital. The use of OLCI and MSI can continue the observation timeline from the MERIS era (2002−2012) using a satellite-to-satellite calibration, leading to an increase in spatial resolution to capture more inland water bodies than previously possible. To reach this point it will be important to know how the atmospheric correction performs at these high resolutions (and lower signal: noise ratios), and how the band ratio algorithms cope with the time delay between individual band acquisitions with MSI. However, the update of existing algorithms to be usable by these newer, higher-resolution satellites is an important part of extending the timeline of MERIS observations and gaining a better understanding of spatial heterogeneity in inland water bodies.

## Credit author statement

MW: Conceptualization, Formal Analysis, Writing - original draft. SS: Conceptualization, Writing - review & editing. NS: Conceptualization, Writing - review & editing.

## Declaration of Competing Interest

The authors declare that they have no known competing financial interests or personal relationships that could have appeared to influence the work reported in this paper.

## Figures and Tables

**Table A:** Statistics generated using 4 datasets and tuning parameters.

	MAD	RMSD	Bias	R	MAPD
D > 5 km	1.20	1.70	0.04	0.80	49.93
D < 5 km	1.28	1.88	−0.40	0.78	63.88
All (D > 5 km)	1.24	1.78	0.04	0.79	52.63
All	1.11	1.61	−0.40	0.80	54.48

**Table B1:** Statistics from per-lake tuning of OC2 algorithm.

	Prior to tuning					After tuning				
	MAD	RMSD	Bias	R	MAPD	MAD	RMSD	Bias	R	n
Victoria	3.04	3.46	−2.94	0.33	59.68	1.25	1.63	−0.08	0.54	1,353,879
Michigan	0.67	1.08	0.15	0.59	128.48	0.4	0.91	−0.18	0.72	503,988
Erie	0.78	1.14	−0.1	0.89	72.26	0.44	0.91	−0.08	0.83	594,125
Titicaca	0.53	1.41	0.2	0.07	78	0.37	0.57	−0.06	0.57	91,122
Turkana	5.59	5.7	−5.43	−0.44	75.68	0.83	1.05	0.02	0.5	31,694
Vattern	0.59	1.08	−0.36	0.19	36.37	0.52	0.87	−0.18	0.45	92,743
Malaren	6.3	9.2	5.47	−0.06	646.61	1.84	2.85	−1.4	0.07	2713
Kinbasket	0.37	0.58	−0.03	0.43	30.11	0.36	0.56	0.01	0.49	5229
Couture	0.26	0.31	0.22	0.4	53.66	0.16	0.21	−0.01	0.51	167
Trout	2.56	3.64	−2.31	0.25	69.18	2.03	2.98	0.1	0.39	2738
Dorsoidong	2.24	3.2	−1.66	0.59	107.62	1.39	2.53	−0.02	0.6	32
Garda	0.35	0.42	0.16	0.63	36.12	0.27	0.39	−0.1	0.6	13,027
Dezadeash	2.33	3.28	1.06	0.04	161.9	1.46	2.44	−1.21	0.08	42
Assean	5.64	6.58	5.27	−0.1	634.81	1.66	2.72	−1.11	0.06	180
Sasyk	1.54	3.26	0.25	−0.02	156.26	1.08	3.77	0.49	0.1	190
Margaret	2.55	3.79	−2.14	0.04	93.5	2.27	3.29	−1.1	0.23	371
Leven	6.76	8.16	6.17	−0.13	578.15	0.63	1.27	−0.39	0.66	27
Lumina	2.86	3.58	−0.3	0.29	158.37	2.57	3.09	−0.22	0.3	80
Isac	3.39	3.8	0.87	0.18	162.67	2.36	3.46	−1.62	0.27	72
Pyhajarvi	4.55	5.64	3.88	0.04	357.39	2.32	2.88	−0.11	0.04	455

**Table B2:** Coefficients from OC2 per-lake tuning.

	a_0_	a_1_	a_2_	a_3_	a_4_
Victoria	0.61	−1.01	8.54	−50.14	−402.4
Michigan	0.33	−5.95	−6.98	113.3	−191.8
Erie	0.28	−5.5	−14.2	53.4	832.85
Titicaca	0.27	−1.67	−14	27.84	104.51
Turkana	1.59	20.96	227.27	975.89	1299.92
Vattern	0.33	−2.78	−2.63	39.77	56.58
Malaren	−0.02	1.08	37.58	187.06	249.08
Kinbasket	0.27	−3.1	−2.52	134.39	271.93
Couture	−6.49	194.71	−2045.77	8991.38	−14,236.58
Trout	0.89	0.84	−72.63	−30.02	1811.47
Dorsoidong	0.82	3.69	0.24	−4658.15	30,462.52
Garda	0.28	2.53	−348.46	4776.79	−18,871.85
Dezadeash	12.18	392.7	4647.26	23,992.51	45,903.59
Assean	0.63	27.69	313.32	1258.44	1668.76
Sasyk	0.36	−4.07	−5.77	−314.46	−1031.95
Margaret	0.03	−8.1	14.03	140.64	−284.9
Leven	8.95	207.98	1627.18	5259.3	6052.77
Lumina	−0.39	−33.13	−366.8	−1482.77	−1928.09
Isac	0.38	11.18	140.43	605.05	913.14
Pyhajarvi	0.3	2.2	−26.9	−322.12	−642.64

**Table C1:** Statistics describing the spread of the bootstrapping results for each algorithm coefficient.

	Mean	Std dev.	Min	25%	50%	75%	Max
OC2: a_0_	0.382	0.0096	0.347	0.376	0.382	0.388	0.419
OC2: a_1_	−4.96	0.134	−5.426	−5.053	−4.964	−4.871	−4.132
OC2: a_2_	−1.017	1.303	−8.601	−1.819	−0.997	−0.209	4.126
OC2: a_3_	57.215	4.783	39.107	53.733	57.386	60.573	72.335
OC2: a_4_	−31.14	24.134	−126.69	−46.045	−31.526	−16.67	102.45
OC3: a_0_	0.312	0.006	0.289	0.308	0.312	0.316	0.336
OC3: a_1_	−1.762	0.059	−2.025	−1.801	−1.761	−1.722	−1.526
OC3: a_2_	2.902	0.443	0.686	2.613	2.912	3.201	4.375
OC3: a_3_	3.416	0.989	0.437	2.760	3.294	3.905	12.276
OC3: a_4_	−28.207	3.533	−41.439	−30.525	−28.359	−26.097	−5.982
Gilerson: a	9.426	1.184	5.351	8.612	9.380	10.192	14.071
Gilerson: b	−3.404	0.659	−6.073	−3.830	−3.376	−2.947	−1.265
Gilerson: c	1.735	0.084	1.491	1.676	1.730	1.788	2.197
Gons05: p	1.066	0.009	1.053	1.060	1.062	1.073	1.105
Gons05: a^⁎^_chl_	0.019	0.0002	0.0183	0.0191	0.0192	0.0193	0.0198

## Appendix A Distance-to-land effects

Tuning results for OC2 from four alternative datasets (Table A). The first row shows the result when tuning using only data where distance to land (D) was greater than 5 km. The statistics in the second row are for tuning when using only data where D < 5 km. The third row shows statistics generated when using all data points but with the tuning parameters from the dataset D > 5 km, and the fourth row shows the statistics generated when tuning the full dataset.

There is no clear best-performing set. Comparing the 3rd and 4th rows, statistics generated using all points, shows a lower MAD and RMSD but higher BIAS and MAPD for the set tuned using all points. As the difference is small it was decided to use the entire dataset as this does not exclude small lakes from further analysis, improving global applicability of the results.
